# Herbivory-associated degradation of tomato trichomes and its impact on biological control of *Aculops lycopersici*

**DOI:** 10.1007/s10493-012-9638-6

**Published:** 2012-12-13

**Authors:** Y. M. van Houten, J. J. Glas, H. Hoogerbrugge, J. Rothe, K. J. F. Bolckmans, S. Simoni, J. van Arkel, J. M. Alba, M. R. Kant, M. W. Sabelis

**Affiliations:** 1 Koppert Biological Systems, Veilingweg 14, Postbus 155, 2650 AD Berkel en Rodenrijs, The Netherlands; 2 IBED, Section Population Biology, University of Amsterdam, Science Park 904, 1098 XH Amsterdam, The Netherlands; 3 CRA-ABP Agricultural Research Council, Centre for Agrobiology and Pedobiology, via Lanciola 12/a, 50125 Florence, Italy

**Keywords:** *Aculops lycopersici*, Tomato russet mite, Glandular trichomes, Plant defense, Predatory mites, *Amblydromalus limonicus*, Enemy-free space

## Abstract

Tomato plants have their leaves, petioles and stems covered with glandular trichomes that protect the plant against two-spotted spider mites and many other herbivorous arthropods, but also hinder searching by phytoseiid mites and other natural enemies of these herbivores. This trichome cover creates competitor-free and enemy-free space for the tomato russet mite (TRM) *Aculops lycopersici* (Acari: Eriophyidae), being so minute that it can seek refuge and feed inbetween the glandular trichomes on tomato cultivars currently used in practice. Indeed, several species of predatory mites tested for biological control of TRM have been reported to feed and reproduce when offered TRM as prey in laboratory experiments, yet in practice these predator species appeared to be unable to prevent TRM outbreaks. Using the phytoseiid mite, *Amblydromalus limonicus*, we found exactly the same, but also obtained evidence for successful establishment of a population of this predatory mite on whole plants that had been previously infested with TRM. This successful establishment may be explained by our observation that the defensive barrier of glandular plant trichomes is literally dropped some time after TRM infestation of the tomato plants: the glandular trichome heads first rapidly develop a brownish discoloration after which they dry out and fall over onto the plant surface. Wherever TRM triggered this response, predatory mites were able to successfully establish a population. Nevertheless, biological control was still unsuccessful because trichome deterioration in TRM-infested areas takes a couple of days to take effect and because it is not a systemic response in the plant, thereby enabling TRM to seek temporary refuge from predation in pest-free trichome-dense areas which continue to be formed while the plant grows. We formulate a hypothesis unifying these observations into one framework with an explicit set of assumptions and predictions to be tested in future experiments.

**Electronic supplementary material:**

The online version of this article (doi:10.1007/s10493-012-9638-6) contains supplementary material, which is available to authorized users.

## Introduction

Eriophyoid mites are among the smallest arthropods on earth (Lindquist et al. 1996). Their worm-like body has a cross-section diameter of c. 50  μm, at least five times smaller than that of phytoseiid mites, several species of which are their most significant predators (Sabelis 1996). The minute size of the eriophyoid mite is the key to their ecological success, enabling them to reach places small enough to be free of predators and still suitable to get access to food resources in the plant (Sabelis and Bruin 1996). Moreover, it allows many of them to develop a plant-parasitic life-style (Lindquist et al. 1996). Many eriophyoids live in plant galls they induce, and others have a vagrant life-style, frequently changing feeding sites that vary in the degree of protection against predators (Sabelis and Bruin 1996; Conijn et al. 1996; Lesna et al. 2005). In agricultural crops, such mites may easily reach pest status when predators are lacking. Chemical control is often ineffective because the eriophyoids may feed under protective structures of the plant (Lindquist et al. 1996). Although such structures can also hamper predators, biological control with predatory mites appeared a promising solution (e.g. Lesna et al. 2005).

Dense covers of (glandular) hairs are known to protect vital plant parts against many herbivorous arthropods (Wagner et al. 2004; Peiffer et al. 2009; Kang et al. 2010). On tomato, glandular trichomes on leaves and stems protect the plant constitutively against two-spotted spider mites (Chatzivasileiadis and Sabelis 1997, 1998; Chatzivasileiadis et al. 1999, 2001; Alba et al. 2009), but they may also hinder predatory mites (Van Haren et al. 1987) and other natural enemies (Simmons and Gurr 2005). In theory, this would create competitor-free and enemy-free space for eriophyoid mites being so minute that they can seek refuge and feed between the glandular hairs. A pest outbreak of such mites would then be inevitable unless tomato plants can readjust their constitutive defenses to prevent them from backfiring (Eisner et al. 1998).

The tomato russet mite *Aculops lycopersici* (Tryon) represents such a case. On tomato, this mite uses its c. 10 μm long stylets to feed on epidermal cells between the glandular trichomes that are present on leaves, but even more so on those of the petioles and stems of tomato. In absence of control measures, this eriophyoid mite becomes a pest (Perring 1996). Efforts to find natural enemies for control of tomato russet mites have resulted in a long list of candidate predators, especially among the Phytoseiidae (e.g. Park et al. 2010). Several species of these predatory mites were found in natural association with tomato russet mites and all of them showed a capacity to feed and reproduce on a diet of tomato russet mites alone. The latter tests were carried out on small tomato leaf discs under laboratory conditions. However, the candidate predators tested for their control capacity on whole plants failed to suppress the pest (Trottin-Caudal et al. 2003; Fischer et al. 2005) or required more generations to adapt to tomato as a host plant (Van Haren et al. 1987) and have an impact on the pest (Castagnoli et al. 2003).

In this article we present similar bioassays for another predatory mite, *Amblydromalus limonicus* (Garman and McGregor), but go further in assessing the underlying causes for success or failure (1) to feed on tomato russet mites (2) to establish a population on a whole plant infested with tomato russet mites and (3) to control TRM on whole plants. Finally (4), we present data showing how TRM modifies plant morphology (in particular that of tomato trichomes) and formulate a testable hypothesis to explain the results on (1)–(3).

## Materials and methods

### Origin of the predator strain and tomato plants

The strain of *A. limonicus* used in our experiments was collected in New Zealand by Peter Workman (Crop and Food Research, Auckland) on beans and tomatoes in 2007. After collection, he predatory mites were reared on a diet of *Typha latifolia* pollen for 1.5 years. The mites for the oviposition test were taken directly from this culture; the mites for the establishment test were reared on tomato plants heavily infested with TRM for 10 weeks before the start of the experiment. The *A. limonicus* used for the population test were reared on astigmatid mites for at least 3 months. All experiments were carried out on tomato (*Solanum lycopersicum*) cv Elanto, with the exception of the direct (microscopical) observations on changes in trichome morphology which were done on tomato (*S. lycopersicum*) cv Castlemart.

**Fig. 1:**
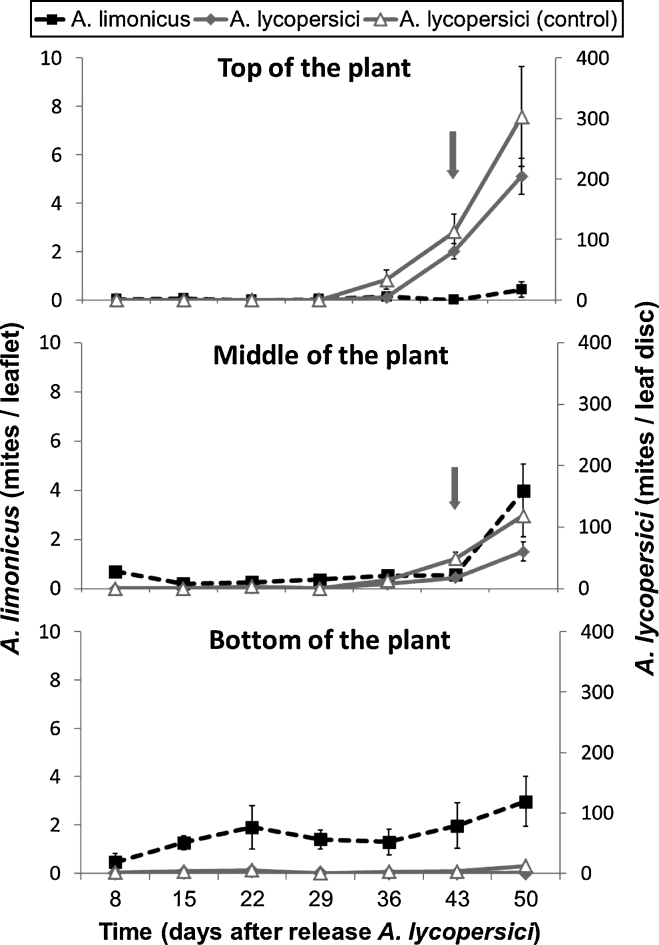
Population dynamics of tomato russet mites (*grey diamonds*) and predatory mites (*Amblydromalus limonicus; black squares*) and of tomato russet mites (*Aculops lycopersici*; *open grey triangles*) in absence of predators in three different strata (*top*, *middle*, *bottom*) of tomato plants over a period of 7 week. 5 leaf discs, each with 280 tomato russet mites, were placed on leaf 3–7 on day 0. Six days later, 140 predatory mites (all mobile stages) were randomly distributed over leaf 3–7. Means are shown as points (*triangles*, *diamonds*, *squares*) and associated vertical bars represent SE. Each of the two treatments was replicated three times. The *vertical arrows* indicate the approximate time period in which glandular hair fall-over takes place on the stems. Differences in TRM population at the last sampling date between plants with and without predators did not test significantly different using the two-sample-test (see “Results” section)

### Oviposition test

To assess the ability of *A. limonicus* to reproduce on TRM, we introduced a young gravid female on a tomato leaf disc (7 cm^2^) with >300 TRM (mixed stages). Over a period of 4 days the predatory mites were transferred each day to fresh leaf disks with TRM, thereby ensuring an ample supply of prey and enabling assessment of the number of predatory mite eggs produced per day. The leaf disc was placed upside down on a layer of agar at the bottom of a small plastic cup. The cup was closed by a lid with a gauze-covered hole to allow air exchange and it was placed in a climate room at 25 °C and 75 % RH under a L16:D8 light regime. To avoid effects of the rearing diet to which the predatory mites had been exposed prior to the experiment, egg production during the first day was excluded from analysis and that during the other 3 days was taken into account. We also repeated exactly the same test with young (<1 day old) whitefly eggs (*Trialeurodes vaporariorum* (Westwood)) as prey because predatory mites produce the same amount of offspring eating these eggs as when eating TRM (see “Results” section: ‘Oviposition test’). This allowed us to uncouple the indirect effect of TRM on the predatory mite via alterations in the host plant and the direct effect of TRM on the predatory mite, being its prey. The data of both experiments were statistically analyzed by a Student *t* test to compare two means from unequal samples and with homogeneous variance.

### Establishment tests on TRM-infested tomato plants

To test predatory mite establishment on whole plants infested with TRM, preliminary experiments were carried out in a greenhouse (mean temperature 21 °C, range 16–35 °C; mean relative humidity 60 %, range 24–85 %) in spring 2009. A cucumber leaf disc (4.5 cm^2^) with 10 young females of *A. limonicus* was introduced on the bottom (=oldest) leaf from each of 6 young (4-leaf stage) tomato plants, each of which had been infested 4 days earlier by placing 5 leaf discs, each with c. 300 TRM (mixed stages), randomly on the 4 leaves of the plant. The plants were together in a screen cage (3 × 1 × 2 m) to reduce the risk of pest infestation from elsewhere. During the subsequent experimental period plants were allowed to develop from 4 to 9 leaves, but thereafter the plant apices were removed thereby maintaining a constant plant size. After 4 weeks 1 leaflet was taken randomly per compound leaf of each plant. The leaflets collected from each plant were put together in a plastic box and next they were inspected one by one under a binocular microscope to count the total number of predators. The same experiment was carried out with tomato plants of the same age infested by greenhouse whiteflies (*T. vaporariorum*) in order to compare the ability of *A. limonicus* to establish itself on plants infested with russet mites with that on plants infested with an unrelated but also suitable herbivore as prey. To establish the whitefly infestation, 90 adult whiteflies were released in a screen cage with 6 tomato plants (thus c. 15 whiteflies per plant), 2 days prior to the introduction of the predatory mites at the bottom leaves. To ensure favourable food conditions for the predators at the moment of their introduction, these tomato plants were also ‘dusted’ with cattail pollen (*T. latifolia*), a suitable alternative food source for many phytoseiid mites, including *A. limonicus*. Data were statistically analysed by a Games-Howell test to compare means derived from groups with unequal sample sizes and with unequal variances.

Based on the information obtained from the first preliminary trial, we repeated the experiment with TRM and *A. limonicus* early in the summer of 2009 (mean temperature 23 °C, range 19–34 °C; mean relative humidity 60 %, range 25–99 %), but now the leaflets sampled from three strata (three leaves at the bottom, three at the middle and three at the top) of each plant were stored in separate boxes. Moreover, not only predatory mites were counted, but also TRM. Finally, the experiments differed in having a larger TRM inoculum (c. 2,500 instead of c. 1,500), a larger number of tomato plants (10 instead of 6) and two sample dates (3 and 4 weeks after predator introduction) instead of only one. To correct for the effect of differences in absolute densities across replications (see large standard errors in the results section: *Establishment tests on TRM-infested plants*), we expressed the population sizes in each stratum as a proportion of the total population found in all samples from a given plant (at the same sampling date).

**Table 1:** Percentage of total population of predatory mites (all stages of *Amblydromalus limonicus*) (mean ± SE) per tomato plant in one of three strata (bottom, middle, top), 3 and 4 weeks since 10 females were released on the leaves at the bottom of a tomato plant that received c. 2,500 tomato russet mites, 4 days before predator introduction

Period	Stratum	Establishment success (%)	
		Tomato russet mites	Predatory mites
3 weeks			
	Top	13.3 ± 2.5	0.0 ± 0.0
	Middle	53.8 ± 3.5	14.8 ± 9.1
	Bottom	32.9 ± 4.8	85.2 ± 9.1
4 weeks			
	Top	58.4 ± 5.1	11.5 ± 2.8
	Middle	35.3 ± 4.6	43.6 ± 7.3
	Bottom	6.2 ± 1.6	44.8 ± 8.2

Number of plants is 10 (=replicates). Data are not statistically analyzed because the differences between mean % TRM and predators in the top stratum (after 3 and 4 weeks) are much larger than 3 times their SE

### Small-scale biocontrol tests

To test for the impact of *A. limonicus* on biological control of TRM, we carried out experiments in 6 screen cages (12 m^2^ ground surface), each with 10 tomato plants, in an experimental greenhouse during the summer of 2009 (mean temperature 23 °C, range 19–35 °C; mean relative humidity 70 %, range 24–99 %). At the start of the experiments the tomato plants, each with 10 fully expanded leaves, received a leaf disc with TRM on the 3rd to the 7th leaf from the oldest one (bottom leaf). These discs had been cut from leaflets picked from a well-infested tomato plant and, based on a sample of thirty leaf discs taken at random, we estimated that each disc harboured on average c. 280 mobile stages of TRM. Each plant received five of these leaf discs (and thus a total of 1,400 TRM). Six days after the introduction of the TRM-discs, c. 140 predators (all mobile stages of *A. limonicus*) per tomato plant were introduced onto leaves 3–7 in 3 randomly selected cages. During the subsequent experimental period plants were allowed to grow until they had 16 leaves, but thereafter plant apices and side-shoots were removed, thereby maintaining a constant plant size and leaf area.

To monitor the temporal population dynamics of TRM and of *A. limonicus* in three strata of the plant, 3 leaflets were taken from each plant (30 leaflets/cage), once a week from day 8 (since TRM introduction) onwards: one leaflet from the lower part (stratum 1: leaf 2–6), one from the middle (stratum 2: leaf 7–11) and one from the top of the plant (Stratum 3: 12–16). The leaflets on which the predatory mites and the TRM had been released were excluded from sampling since we wanted to assess arthropod mobility across strata during the course of the experiment. First, only the predatory mites on each leaflet sampled per stratum were counted under a stereomicroscope, because they are relatively agile. Next, the number of TRM was assessed by counting their mobile stages in a representative subsample, i.e. small leaf discs (3.5 cm^2^), each cut from an additional leaflet obtained from the same strata from which we had assessed the predatory mite densities.

### Assessments of tomato trichome deterioration in response to TRM infestation

Using a stereomicroscope equipped with a photo camera, we inspected (at 30–40× magnification) trichomes of the glandular (Type I and VI) and non-glandular (Type III and V) type on leaves and stems of TRM-infested tomato plants as well as TRM-free plants (Luckwill 1943). Visible changes in external appearance of the trichomes and the whole plant were recorded on photograph at least once every 3–4 days. The results of these observations are summarized in a video providing a slideshow of the morphological and other changes in trichomes of TRM-infested tomato leaves, petioles and stems (supplementary material).

To quantify the process of trichome deterioration trichomes were counted on three plants (3 weeks-old) infested with russet mites (c. 1,500 mixed stages) and on three uninfested control plants at the basis of the main stem. Three stem/petiole sections (6 mm in length) were studied in detail, one in the middle of the main stem, one on the petiole of the first true leaf (petiole 1) and one on the petiole of the second leaf (petiole 2) (leaves are numbered from the bottom of the plant). Photographs (taken against a contrasting background) were taken of these sections and then used to count intact (=unaffected) and amber-coloured Type VI trichomes (Luckwill 1943) (to facilitate counting this was done in each of three consecutive subsections of 2 mm in length). This procedure was carried out on day 0, 4, 7, 11, 14 and 18 after TRM infestation.

**Fig. 2:**
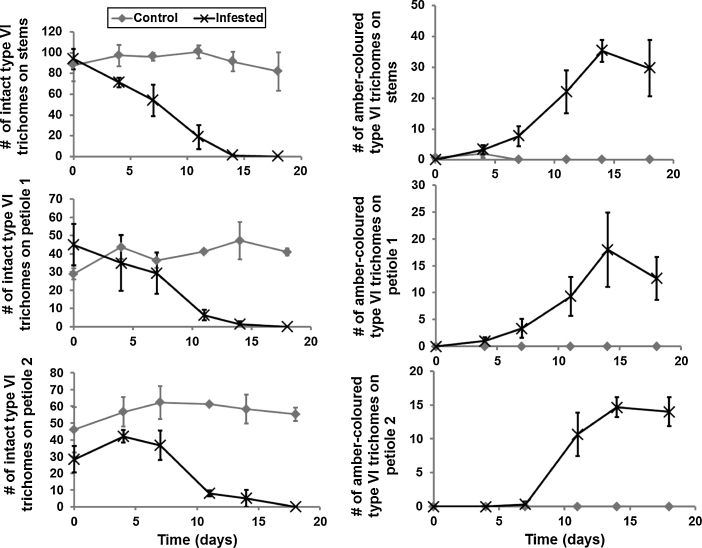
Changes in the number of intact Type VI trichomes (*left panels*) and amber-coloured Type VI trichomes (*right panels*) over a period of 18 days. Data represent means and standard errors of the number of trichomes per section (6 mm in length) per plant. *Upper panels* relate to main stem sections, *middle panels* to petiole 1 sections and *lower panels* to petiole 2 sections. For the number of intact type VI trichomes on stems the lines start to separate significantly on day 4; on petiole 1 on day 11 and on petiole 2 on day 11. Moreover, trichome ambering became apparent on the stems on day 7; on petiole 1 on day 7 and on petiole 2 on day 10 (*t* test, α = 0.05)

## Results

### Oviposition test

The oviposition rate of young *A. limonicus* females fed on a diet of (amply supplied) TRM (all stages) did not significantly differ from that on a diet of (amply supplied) greenhouse whitefly eggs. Young adult females of the predatory mite *A. limonicus* produced on tomato leaf discs with fresh whitefly eggs as food (n = 13) on average 3.0 ± 0.1 eggs and on discs with tomato russet mites (all stages; n = 8) as food on average 3.6 ± 0.2 eggs. These mean oviposition rates are not significantly different according to a *t* test with unequal sample sizes, assuming homogeneous variances (t = 1.095; *p* > 0.2). Thus, if these two prey-diets, i.e. their quality and/or quantity, were the only determinants of predator establishment, we would expect equal establishment success of *A. limonicus* on tomato plants infested by TRM and those infested by greenhouse whiteflies.

### Establishment tests on TRM-infested plants

In contrast to the above expectation, the first series of preliminary experiments on establishment after 4 weeks showed a significantly higher number of predators on TRM-infested plants than on whitefly-infested plants On plants with whiteflies + *Typha* pollen numbers of predatory mites reached up to 75.7 ± 32.1 adult mites per plant while on plants with TRM it reached levels up to 1,274.0 ± 108.2 adult mites per plant within a time span of 2 weeks. Mean numbers of predators per plant were significantly different according to a Games–Howell test with unequal sample sizes and heterogeneous variances (since the minimum significant difference MSD at the 5 % level equals 47.38 which is much less than the difference between means).

Although prey densities had not been precisely quantified in the latter experiments, they can be considered to represent an ample prey supply to achieve maximum reproduction. Thus, we had no reason to assume that the observed difference in establishment success was due to the amount of prey (and pollen as alternative food) present on these plants.

We also carried out a second series of experiments on predatory mite establishment, differing from the first in that samples of predatory mites, as well as TRMs, were taken from different strata in the plant. The results, given as mean and SE of the percentages per plant (Table 1), did not require statistical treatment because the variables of interest, i.e. the proportions of the (TRM and predator) population we recorded in the top stratum, differed more than 3 times their SE and suggesting that the TRM populations spread much faster from the lowest stratum (where they were introduced on the plant) to the top stratum, than do the predatory mites (also released at the bottom of the plant). Direct observations on the tomato plants showed that progress of the predatory mites to upper parts in the plant was strongly synchronized with the change in trichome density due to their collapse.

### Small-scale biocontrol test

As a final test, replicated experiments on biocontrol of TRM with the predatory mite *A. limonicus* and on TRM dynamics without predators showed that the bottom stratum of the plant harbours very few TRM and that the predator population stabilizes rapidly, but is much smaller than originally released (c. 2 %) (Fig. 1; lower panel). The middle stratum shows an initial increase of the TRM population during the last week and this is slightly stronger in absence than in presence of the predators (Fig. 1; middle panel). The predators were hardly found in the middle stratum until in the last week of the experiment. In the top stratum the TRM population increased strongly during the last 3 weeks and this was clearly strongest in absence of the predators (Fig. 1; top panel). Predators were rarely ever found in the top stratum and only very few individuals appeared in the last week of the experiment. We conclude that, even though there was little or no prey at the bottom of the plant, we had not recorded any predators that had moved up to the higher strata until the last week of the experiment, whereas the TRM population readily spread to the top of the plant where their populations build up starting from 3 weeks before the end of the experiment. Overall, there is reason to think that the predator population had some suppressive effect on the growth rate of the TRM population, but biocontrol was clearly ineffective. Differences in TRM population at the last sampling date between plants with and without predators were not significant [two-sample-test: *p* = 0.38 (top), *p* = 0.26 (middle), *p* = 0.12 (bottom), *p* = 0.27 (overall)].

### Assessments of trichome deterioration in response to TRM infestation

A possible explanation for the differential establishment success emerged from direct observations on the plants in this experiment. We noticed a striking response of tomato plants to TRM but not to whiteflies: during TRM-infestation first the colour in the glandular heads changed from oblique to amber-coloured, followed by drying out of the stalk and finally rapid deterioration of both glandular and non-glandular trichomes altogether. Massive trichome collapse could be observed wherever TRM numbers increased beyond a threshold of c. 50 mobiles per cm^2^ which is a density easily reached within days after a moderate starting infection. Trichome collapse, especially that of the glandular trichomes, may remove a barrier critical to the establishment of predatory mites. Detailed observations on changes in the trichomes (see supplementary material with video providing slideshow) showed that (1) trichome collapse only occurs on plant parts with TRM but not systemically, that (2) non-glandular trichomes deteriorate within 3 days, that (3) glandular trichomes turn amber-coloured after 3 days and that (4) they collapse within a week, depending on the density of TRM. Thus, the sampling time intervals selected in the experiments to follow (3–4 weeks in the establishment test; 7 weeks in the biocontrol test) are long enough to get severe trichome deterioration.

Quantitative assessments of Type VI trichomes on stems and petioles of TRM-infested tomato plants showed a gradual decline in intact trichomes and a gradual increase in amber-coloured trichomes over a period of 18 days (Fig. 2). Since trichome collapse is a progressive phenotype and the time points are dependent data we only determined the time point at which the control and treatment line (Fig 2) numerically start separating using a *t* test on the individual time points at α ≤ 0.05. For the number of intact type VI trichomes on stems the lines start to separate significantly at day 4; on petiole 1 on day 11 and on petiole 2 on day 11. Moreover, trichome ambering became apparent on the stems on day 7; on petiole 1 on day 7 and on petiole 2 on day 10. However, uninfested plants (with or without apex/leaf removal) do not show trichome deterioration at all. This confirms the visual observations in the experiments on predator establishment and TRM biocontrol.

## Discussion

As reported for several other phytoseiid mites (see introduction), the generalist predatory mite, *A. limonicus*, can feed and reproduce on a diet of TRM alone when offered on tomato leaves with low trichome densities. This explains why our preliminary trials indicated that the predatory mite was able to establish and rapidly build up a population on whole tomato plants heavily infested with TRM. However, despite successful establishment this predator was *not* able to control TRM on whole plants inoculated with TRM. Possibly releasing more predatory mites, or more frequently, could improve their capacity to control TRM. Nevertheless, in the presence of an initially high and expanding population of predatory mites, the TRM population continued to increase, albeit at a slower pace (probably due to predation). In the course of the biocontrol experiment we observed that over time there was a gradual decrease in active glandular trichomes on leaves, petioles and stems because they dried out and collapsed. This process required days rather than hours and became apparent only after TRM had developed a population for several days up to a week.

We hypothesize that the ineffective control by the predatory mites arises because the TRM populations escaped predator control by moving up in the plant at a rate faster than their predators. The relatively slow upward movement of the predators in the plant cannot be due to arrestment of the predators in the lowest strata because here the leaves had very low TRM densities (and no other prey/food) during the last weeks of the experiment. Hence, there must be another factor impeding their spread over the plant. Although in general the reasons for residing in a particular stratum of the plant can be many and varied (e.g. abiotic conditions in the mid-stratum as argued by Shipp and Wang (2003) or in the apex as argued by Onzo et al. 2010), our experience with *A. limonicus* (and other phytoseiids) on trichome-free plants is that they stay, or now and then move to, wherever the prey is positioned on the plant (e.g. see also Weintraub et al. 2007; Onzo et al. 2009). Hence we hypothesize that this delay in predator response to TRM movement in the tomato plant arises from the time required for TRM to reach densities sufficiently high to give rise to the collapse of tomato trichomes.

The collapse of glandular and non-glandular trichomes could either be the result of mites directly feeding on the trichomes or from mite-induced physiological changes in the plant. Preliminary data suggest that russet mites suppress JA defences in tomato (Glas and Kant, unpublished data) reminiscent of some spider mite genotypes (Kant et al. 2008; Sarmento et al. 2011). It is well known that a plant’s jasmonate metabolism can have a profound impact on trichomes (Pauwels and Goossens 2011), i.e. on their development (Traw and Bergelson 2003), density (Boughton et al. 2005) and chemistry (Li et al. 2004). The amber discoloration of the glandular trichome heads that precedes their massive collapse (this paper) was also observed during trichome hyperplasia in *Quercus ilex* trichomes upon attack by *Aceria ilicis* mites. Here, the discoloration was paralleled by an increased accumulation of simple phenolics and flavonoids within these trichomes (Karioti et al. 2011). Hence, possibly the amber discoloration is due to accumulation of (oxidized) proanthocyanidins and/or their polymers (Zhao et al. 2010). However, if such changes can be the cause of both glandular and non-glandular trichome deterioration or whether these are only symptomatic of it, remains a question to be elucidated.

Trichome collapse, especially that of the glandular trichomes, may remove a barrier critical to the establishment of predatory mites. This may take effect especially when they have to move from leaf to leaf because tomato cultivars commonly used in practice exhibit the highest densities of glandular trichomes on the stems (Van Haren et al. 1987; Sato et al. 2011). However, TRM is too small to be hindered by tomato trichomes and whitefly nymphs hardly move over the plant surface whereas their adults can fly. Most notably, trichome collapse did not occur all over the surface of the plant, but only where TRM reached sufficiently high densities. Thus, these pest arthropods are not hindered by the glandular trichome ‘forests’ on stems of tomato plants. Moreover, the predatory mites under study established best and built up a (6 times larger) population where the surface of the tomato plants was cleared from glandular trichomes. Taken together this may explain why *A. limonicus* is able to establish on TRM-infested tomato plants but not on whitefly infested plants and/or plants supplied with pollen. Based on the experimental results and direct observations we formulate a hypothesis in an attempt to explain them by one unifying principle. We propose that (1) forests of glandular trichomes, i.e. where they occur at high densities on the plant surface, provide a refuge to TRM by protecting them against predatory mites, that (2) TRM feeding induces the plant to deteriorate trichomes (or alternatively, TRM directly causes trichomes to fall-over), that (3) this allows predatory mites to move on the plant surface unhindered by glandular trichomes, thereby enabling them to find and consume TRM, and that (4) the process of trichome fall-over is local and proceeds slow enough to provide TRM with enemy-free space elsewhere on the plant, thereby making biocontrol with predatory mites unsuccessful. The first conjecture is supported by the observation that the per capita rate of predation on TRM on a plant surface devoid of glandular trichomes is much higher than that on a plant surface with these trichomes, and that TRM tends to seek refuge in the trichome forest especially when there are predatory mites around (Simoni and Sabelis 2010). Note, however, that the very high densities of glandular trichomes found on wild type tomato can provide resistance against TRM (Leite et al. 1999), which could explain why natural selection did not favour TRM genotypes that keep trichomes intact. The second conjecture requires in-depth plant physiological research. From a functional point of view (*sensu* Tinbergen 1963), the plant benefits from trichome deterioration because TRM becomes more exposed to predation provided these predators are around (whereas it would otherwise be destroyed by TRM). Moreover, TRM may also experience direct negative effects from glandular secretions (see e.g. Wagner et al. 2004; Shepherd et al. 2005) or from contact with glandular trichome heads (Peiffer et al. 2009). If so, TRM may attempt to reduce this by disturbing trichome functioning and integrity, but we have no observations so far to substantiate this. The third conjecture is actually the one most strongly supported. Establishment of phytoseiid mites on TRM-infested tomato has been observed not only in the experiments with *A. limonicus* presented in this article, but also in experiments with *Amblyseius swirskii* (Athias-Henriot) and with *Typhlodromips montdorensis* (Schicha) (Van Houten, pers obs.). The fourth conjecture requires experiments to assess the rate of trichome collapse during the ontogeny of a tomato plant in response to TRM feeding initiated on leaves of different age. Also, it would be of interest to find tomato genotypes that exhibit systemic induction of trichome deterioration in response to TRM attack.

We emphasize that our hypothesis requires extensive and detailed experiments before it can be accepted or rejected. Our motivation to formulate this hypothesis is that—by making it explicit—it may stimulate critical tests and thereby advance our insight into the underlying mechanisms determining biocontrol successes or failures on plants with glandular trichomes, such as tomato.

## Electronic supplementary material

Below is the link to the electronic supplementary material.

Supplementary material 1 (MP4 5725 kb)
